# Detection of polymyxin-resistant *Enterobacteriaceae* from poultry farms in Brazil: continued *mcr* gene dissemination

**DOI:** 10.1007/s42770-026-01890-3

**Published:** 2026-03-18

**Authors:** Bruno Rocha Pribul, Kathelyn Soares dos Santos, Ramon Pimenta, Orlando Carlos da Conceição-Neto, Ana Paula D’Alincourt Carvalho-Assef, Miliane Moreira Soares de Souza, Cláudio Marcos Rocha-de-Souza

**Affiliations:** 1 Laboratório de Bacteriologia Aplicada à Saúde Única e Resistência Antimicrobiana (LabSUR), Instituto Oswaldo Cruz (IOC), Fundação Oswaldo Cruz (Fiocruz), Rio de Janeiro, Brazil; 2 Coleção de Culturas de bactérias de origem Hospitalar (CCBH), Instituto Oswaldo Cruz (IOC), Fundação Oswaldo Cruz (Fiocruz), Rio de Janeiro, Brazil; 3 Departamento de Microbiologia e Imunologia Veterinária, Universidade Federal Rural do Rio de Janeiro - UFRRJ, Rio de Janeiro, Brazil; 4 Chefe da subdivisão de Análises Clínicas do Hospital Central da Aeronáutica, Professor Assistente da Faculdade de Medicina da Universidade Estácio de Sá – IDOMED, Rio de Janeiro, 4036 Brasil; 5 Fundação Oswaldo Cruz – Maré. Centro de Pesquisa, Inovação e Vigilância em COVID-19 e Emergências Sanitária, Rio de Janeiro, Brasil

**Keywords:** polymyxin resistance, mcr-1, multidrug resistance bacteria, plasmid transfer, poultry

## Abstract

The emergence and persistence of plasmid-mediated polymyxin resistance in Brazilian poultry production pose a significant One Health challenge. Here, cloacal swabs from 202 broilers across four farms in the State of Rio de Janeiro yielded 125 *Enterobacteriaceae* isolates growing on polymyxin-EMB agar. *Escherichia coli* accounted for 99% of resistant isolates, with one *Klebsiella pneumoniae*. Multidrug resistance (MDR) was observed in 75% of polymyxin-resistant strains. PCR screening revealed *mcr-1* and *mcr-5* genes. Conjugation assays demonstrated horizontal transfer of *mcr-1* plasmids (48.5–194 kb). MLST assigned key strains to ST10 and ST48, both within the high-risk CC10 lineage. These findings underscore the entrenched nature of polymyxin resistance despite regulatory bans, highlight the risk of zoonotic transmission of MDR determinants, and call for enhanced surveillance, biosecurity and alternative interventions to mitigate the spread of mobile polymyxin resistance in poultry environments.

## Introduction

Antimicrobial resistance (AMR) has emerged as one of the most critical challenges in global public health, affecting not only human medicine but also veterinary and agricultural sectors [1, 2].

Poultry production holds a particularly important role in the context of AMR. In Brazil, one of the world’s leading poultry exporters, intensive production systems and the historical use of antibiotics, including those deemed critical for human medicine, have contributed to the selection and maintenance of resistant bacterial populations in poultry flocks [3].

*Escherichia coli*, a commensal bacterium in the avian intestinal tract, can acquire virulence and resistance traits, becoming an important pathogen for both poultry and humans [4]. The emergence of multidrug resistant (MDR) *E. coli*, defined as resistance to at least one agent in three or more different [5], as well as resistance in other opportunistic members of the *Enterobacteriaceae*, raises concern for food safety and zoonotic transmission [6].

Polymyxins have regained importance as last-resort antibiotics for treating Gram-negative infections in humans, especially cases caused by carbapenem-resistant bacteria [7]. Despite their clinical value, polymyxin has been widely used in animal agriculture for both prophylactic and growth-promoting purposes, fuelling the appearance of resistant isolates in livestock [8]. In 2016, the Brazilian Ministry of Agriculture and Livestock banned colistin as a feed additive after the global detection of transferable plasmid-mediated colistin resistance genes (*mcr*) in both animal and human isolates [3].

Since the first description of *mcr-1* gene in *E. coli*, multiple variants (*mcr-1* to *mcr-10*) have been reported spreading across different continents and bacterial species, with poultry production emerging as a primary reservoir and vector of dissemination [9, 10].

Current evidence highlights the persistence of polymyxin and multidrug resistance among poultry associated bacteria even after regulatory actions [11, 12]. The presence of these determinants in food producing animals increases the risk of transmission to humans through multiple pathways. Direct transmission can occur via occupational exposure and the food chain, while indirect transmission may result from environmental contamination. These findings underscore the critical importance of implementing coordinated surveillance strategies across multiple sectors [13].

This study aimed to investigate the prevalence, clonal diversity, antimicrobial susceptibility, and molecular determinants of *mcr*-mediated polymyxin resistance among *Enterobacteriaceae* isolates from poultry farms.

## Materials and methods

### Study design and sample collection

**Fig. 1:**
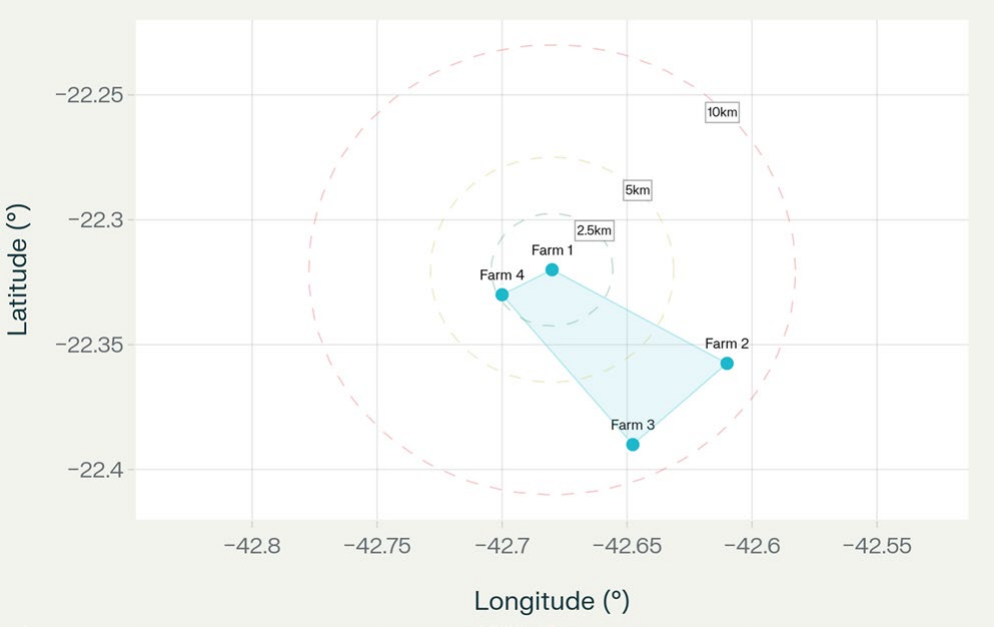
Geographic location of poultry farms in São José do Vale do Rio Preto, Rio de Janeiro State, Brazil (22°21′27″ S, 42°38′52″ W). Four farms were sampled between July 2021 and March 2022: three commercial broiler farms (Farms 1–3) and one free-range laying hen farm (Farm 4). Distance measurements in kilometers are shown between farm locations. Scale bar: 10 km. Note: The number of swabs collected in each farm was: Farm A (*n* = 60), Farm B (*n* = 60), Farm C (*n* = 50), and Farm D (*n* = 32)

Between July 2021 and March 2022, cloacal swabs were collected from chickens across four poultry farms located in São José do Vale do Rio Preto, State of Rio de Janeiro, Brazil (22°21′27″ S, 42°38′52″ W) (Fig. 1). The study comprised three commercial broiler farms and one free-range laying hen farm. Cloacal swabs were collected from each individual, placed in Cary Blair transport medium, kept refrigerated, and processed within 48 h. Sample collection was conducted in collaboration with the Department of Veterinary Microbiology and Immunology at the Federal Rural University of Rio de Janeiro (UFRRJ) under ethical approval CEUA nº 6,239,180,418 (CEUA/UFRRJ).

## Polymyxin-resistant screening and bacterial identification

To screen polymyxin-resistant isolates, swabs were streaked onto Eosin Methylene Blue (EMB) agar (Oxoid, UK) supplemented with 4 µg/mL polymyxin sulphate (Sigma-Aldrich, USA), following procedures commonly applied in studies of colistin/polymyxin resistance surveillance. The use of EMB with polymyxin as a selective platform is supported by prior studies evaluating polymyxin-resistant strains in Brazil [14]. Quality control strains included *Escherichia coli* ATCC 25,922 (polymyxin-susceptible), *Proteus mirabilis* CCBH 22,517 (intrinsically polymyxin-resistant), and *E. coli* CCBH 23,595 (polymyxin-resistant, MIC 4 µg/mL, *mcr-1*-positive). CCBH samples were obtained from the Culture Collection of Hospital-Acquired Bacteria (CCBH), registered with the World Federation for Culture Collections (WFCC, WDCM 947). Isolates demonstrating growth on selective medium were identified using Matrix-Assisted Laser Desorption Ionisation Time-of-Flight Mass Spectrometry (MALDI-TOF MS) (Bruker Daltonics, Germany).

## Antimicrobial Susceptibility Testing

Polymyxin resistance was confirmed using broth microdilution with the commercial Policimbac^®^ test (Probac do Brasil), according to the manufacturer’s instructions. Minimum inhibitory concentration (MIC) values > 2 µg/mL were categorised as resistant, in accordance with BrCAST/EUCAST guidelines (http://www.eucast.org).

Antimicrobial susceptibility to 18 agents was assessed using disc diffusion method using the following antimicrobial agents: ampicillin (10 µg), cefoxitin (30 µg), cefuroxime (30 µg), ceftazidime (10 µg), ceftriaxone (30 µg), cefepime (30 µg), ertapenem (10 µg), imipenem (10 µg), meropenem (10 µg), amikacin (30 µg), gentamicin (10 µg), nalidixic acid (30 µg), ciprofloxacin (5 µg), tetracycline (30 µg), chloramphenicol (30 µg), tigecycline (15 µg), aztreonam (30 µg), trimethoprim–sulfamethoxazole (25 µg). Results were interpreted according to BrCAST 2025 criteria, except for nalidixic acid, which was evaluated based on the Clinical and Laboratory Standards Institute (CLSI) M100 guidelines.

### PCR Screening for *mcr* genes

Multiplex PCR for detection of *mcr* genes was performed following the validated EURL/DTU protocol [15]. Reaction composition for a 25 µL reaction: DreamTaq Green PCR Master Mix (2×) 12.5 µL; primer mix5 µL (primer mix prepared so that each primer stock is 10 µM and final primer concentration as per the EURL protocol); DNA template 2 µL; nuclease-free water to 25 µL. Thermocycling: 94 °C 15 min; 25 cycles of (94 °C 30 s, 58 °C 90 s, 72 °C 60 s); final extension 72 °C 10 min. Amplification products were visualized by agarose gel electrophoresis. Controls included *mcr*-positive controls for *mcr*-1, *mcr*-5 and *mcr*-9: *E. coli* CCBH20180 (*mcr*-1-positive), *E. coli* CCBH25606 (*mcr*-5-positive), and *E. coli* CCBH27304 (*mcr*-9-positive). The primer sequences were as follows: *mcr-1* forward 5’-AGTCCGTTTGTTCTTGTGGC-3’, reverse 5’-AGATCCTTGGTCTCGGCTTG-3’; *mcr-5* forward 5’-ATGCGGTTGTCTGCATTTATC-3’, reverse 5’-TCATTGTGGTTGTCCTTTTCTG-3’; *mcr-9* forward 5’-GTATCCTTCCTGCCATCCTC-3’, reverse 5’-CTTTCCATAACAGCGAGACAC-3’.

## Conjugation Assays

Horizontal transfer capability of *mcr*-carrying plasmids was evaluated using conjugation experiments. The experiments were conducted using the filter-mating method. Donor *mcr-1*-harboring strains (polymyxin-resistant isolates) *E. coli* 2 A (Farm 1) / *K. pneumoniae* 11 F (Farm 2) and the *E. coli* J53 recipient (sodium azide–resistant) were grown to mid-log phase in MH broth, mixed at a 1:1 ratio, and spotted onto nitrocellulose membranes placed on MH agar plates. After incubation at 37 °C for 18–20 h, the bacterial growth was resuspended in 1 mL of saline solution, serially diluted, and plated onto selective MH agar containing sodium azide (200 µg/mL) and polymyxin sulphate (4.0 µg/mL) to recover transconjugants. Presumptive transconjugants were confirmed by PCR amplification of the *mcr-1* gene and by species identification using MALDI-TOF MS. This procedure follows validated filter-mating protocols commonly used for evaluating the transferability of *mcr*-mediated resistance plasmids [16, 17].

## Genetic Diversity Analysis

Clonal relationships among *E. coli* isolates were assessed using pulsed-field gel electrophoresis (PFGE) with XbaI restriction enzyme following established protocols [18]. DNA fragments were separated using the CHEF-DRIII system (Bio-Rad, USA) under optimized conditions, and dendrograms were constructed using BioNumerics 6.6 software with Dice coefficient analysis, where isolates sharing ≥ 80% similarity were considered members of the same clonal group. Plasmid characterization was performed using S1-nuclease PFGE to determine the molecular weights and number of plasmids in both clinical isolates and transconjugants.

Multilocus sequence typing (MLST) was performed using 7 housekeeping genes (*adk*, *fumC*, *gyrB*, *icd*, *mdh*, *purA*, *recA*), using standard primers and PCR conditions recommended by the MLST consortium. Amplicons were purified and sequenced by the Sanger method. Forward and reverse reads were assembled using BioEdit and submitted to the *E. coli* MLST database (https://pubmlst.org/) for allele assignment. Sequence types (STs) were defined according to the corresponding allele profiles. Only isolates with high-quality bidirectional sequence coverage were assigned STs.”

### Statistical Analysis

Resistance frequency for each farm was calculated as the proportion of resistant isolates with 95% confidence intervals (CI) computed using the Clopper-Pearson exact binomial method, which provides accurate confidence limits for proportions regardless of sample size [19]. These values represent the observed frequencies within the sampled population, rather than estimates of population prevalence, given that the sampling design was not probabilistic.

## Results

### Sample Collection and Bacterial Isolation

A total of 202 cloacal swabs were collected from 4 poultry farms in the State of Rio de Janeiro, comprising 3 broiler establishments and 1 free-range laying farm. The distribution of cloacal swabs per farm was as follows: Farm A (*n* = 60), Farm B (*n* = 60), Farm C (*n* = 50), and Farm D (*n* = 32). Among the collected samples, 125 bacterial isolates exhibited growth on EMB agar (4 µg/mL polymyxin). Most of these isolates came from broiler farm 1 (45.6%, 57/125), followed by farm 3 (28%, 35/125), farm 2 (23.2%, 29/125) and the laying farm 4 (3.2%, 4/125). Species identification by MALDI-TOF MS revealed that 99% (124/125) of isolates were *E. coli*, with one isolate (1%) identified as *Klebsiella pneumoniae*.

### Polymyxin resistance patterns and antimicrobial susceptibility profiles

**Fig. 2:**
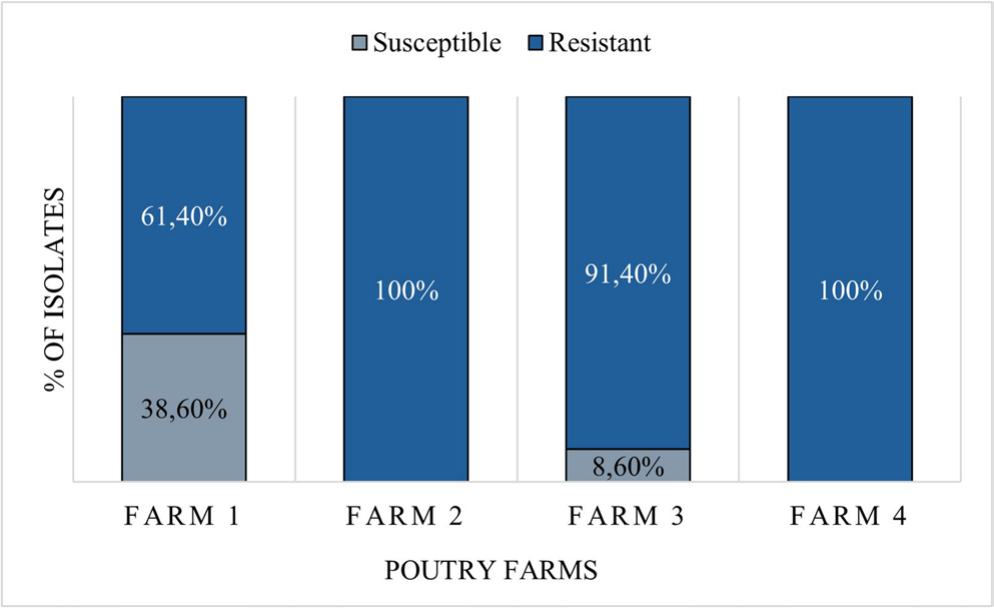
The chart illustrates polymyxin resistance distribution across four poultry farms, created using Microsoft Excel 365. Proportion of susceptible (grey) and resistant (blue) *Enterobacteriaceae* isolates determined by broth microdilution. Resistance rates varied from 38.6% (Farm 1) to 100% (Farms 2 and 4), with Farm 3 showing 91.4% resistance. Overall resistance frequency was 69.6% (87/125 isolates; 95% CI 60.4–77.4%) comprising 86 *E. coli* and 1 *K. pneumoniae*

Broth microdilution confirmed polymyxin resistance in 87 of 125 cloacal-swab isolates (69.6%; 95% CI 60.4–77.4%), comprising 86 *E. coli* and 1 *K. pneumoniae*. MICs ranged from 8 to 64 µg/mL (MIC₅₀ = 16 µg/mL; MIC₉₀ = 32 µg/mL). Resistance frequency varied by farm: 38.6% (*n* = 22; 95% CI 27.1–51.6%) at Farm 1, 100% (*n* = 29; 95% CI 88.3–100%) at Farm 2, 91.4% (*n* = 32; 95% CI 77.6–97.0%) at Farm 3, and 100% (*n* = 4; 95% CI 51.0–100%) at Farm 4 (Fig. 2).

**Fig. 3:**
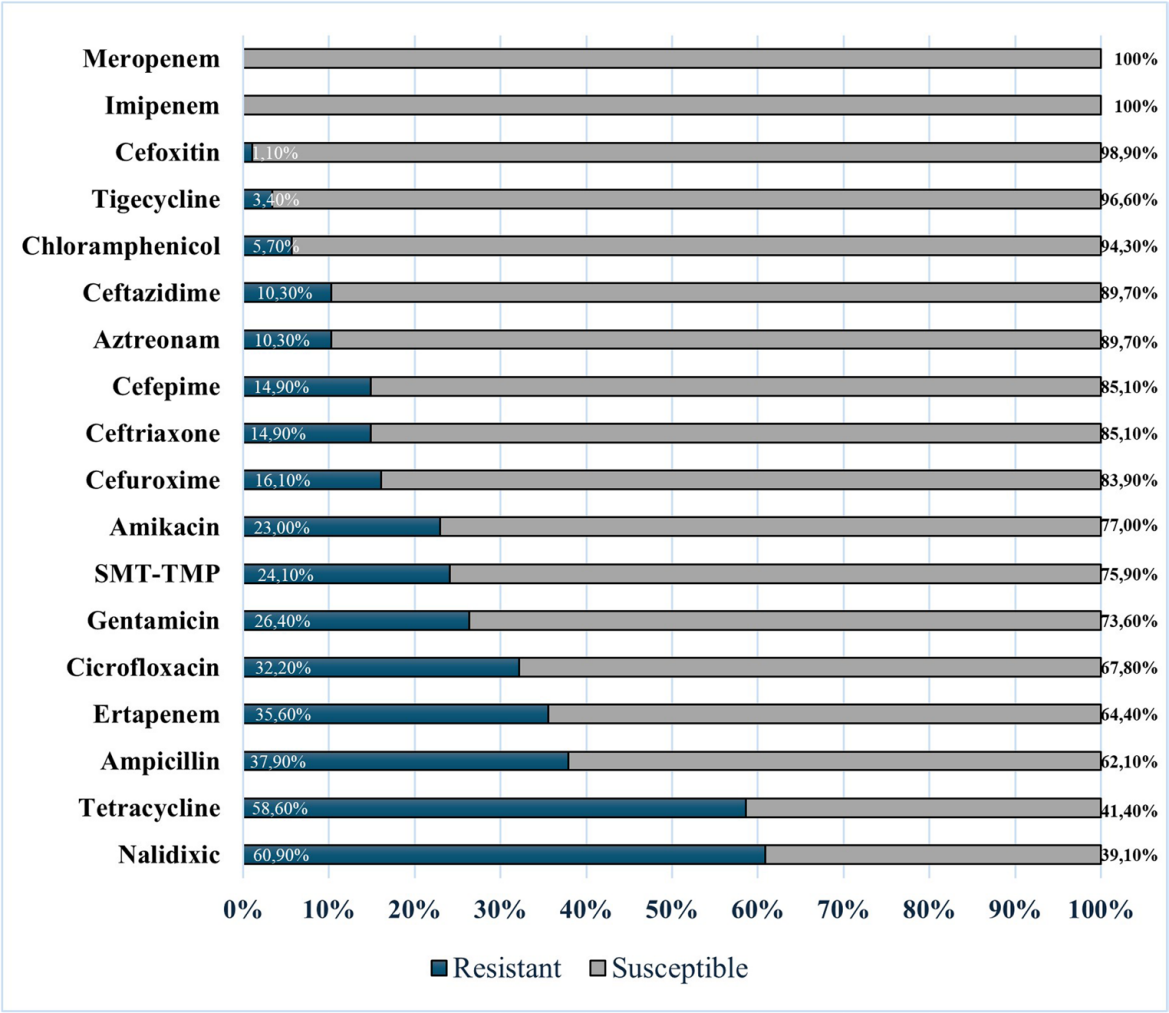
Antimicrobial resistance profiles of polymyxin-resistant *Enterobacteriaceae* isolates from poultry farms, created using Microsoft Excel 365. Horizontal stacked bar chart displaying resistance (dark blue) and susceptibility (grey) percentages across 18 antimicrobial agents tested against 87 isolates (86 *E. coli*, 1 *K. pneumoniae*). Highest resistance rates were observed for nalidixic acid (60.9%) and tetracycline (58.6%), whilst complete susceptibility was maintained to carbapenems (imipenem and meropenem, 100% susceptible). Note: Antibiogram percentages refer to the 87 polymyxin-resistant isolates (86 *E. coli* and 1 *K. pneumoniae*). As *Klebsiella pneumoniae* is intrinsically resistant to ampicillin, we note that excluding the single *K. pneumoniae* from the denominator changes the reported ampicillin resistance from 37.9% to 37.2% (86 *E. coli* only).

Antimicrobial susceptibility testing revealed marked heterogeneity in resistance patterns amongst the 87 isolates examined. The highest resistance rates, considering the 87 isolates resistant to polymyxin (*E. coli*, *n* = 86; *K. pneumoniae*, *n* = 86), were observed for nalidixic acid (60.9%) and tetracycline (58.6%), followed by ampicillin (37.9%), ertapenem (35.6%), and ciprofloxacin (32.2%). Intermediate resistance frequencies were recorded for gentamicin (26.4%), sulfamethoxazole-trimethoprim (24.1%), amikacin (23.0%), cefuroxime (16.1%), cefepime and ceftriaxone (both 14.9%), and ceftazidime and aztreonam (both 10.3%). The lowest resistance rates were demonstrated against chloramphenicol (5.7%), tigecycline (3.4%), and cefoxitin (1.1%). Notably, all isolates remained fully susceptible to imipenem and meropenem (Fig. 3).

Multidrug resistance (MDR) was defined as non-susceptibility to at least one agent in three or more antimicrobial classes [20]. In line with this definition, antimicrobial classes were categorised according to the framework proposed by Magiorakos et al. (2012), which treats β-lactam subclasses (penicillins, cephalosporins, monobactams, and carbapenems) as independent epidemiological categories due to their distinct resistance mechanisms. Accordingly, each subclass was considered separately when determining MDR profiles, consistent with the 15-category structure of the Magiorakos classification. These criteria were applied uniformly to all isolates and, according to them, 75% (66/87) were classified as MDR. These 66 *E. coli* strains were grouped into 36 distinct resistance profiles based on the combination of classes to which they were non-susceptible. Two isolates were assigned to the same profile when they showed identical patterns of non-susceptibility in all antimicrobial classes tested. The most common profiles (P27, P32 and P33) each comprised 6 isolates. Profile P27 (quinolones-tetracyclines-carbapenems-polymyxin) was detected predominantly on farm 2, profile P32 (penicillins-tetracyclines-polymyxin) was most frequent on farm 3, and profile P33 (quinolones-carbapenems-polymyxin) occurred mainly on farm 1. Among the MDR isolates, 34.8% were resistant to three classes, 36.4% to four classes, 12.1% to five classes, 6.1% to six classes, 6.1% to seven classes and 4.5% to eight classes. This distribution illustrates the varied and farm-specific multidrug resistance patterns circulating within the poultry farms surveyed (Table 1).

**Table 1:** Multidrug resistance profiles determined for 66 *Escherichia coli* isolates

Profile	No. of non-susceptible classes	Non-susceptible classes/categories	polymyxin-*R* E. coli, *n*
P1	8	PEN/ AMG/ CEF/ TET/ ANF/ IVF/ MON/ POL	1
P2	8	PEN/ AMG/ CEF/ TET/GLI/ IVF/MON/ POL	1
P3	8	PEN/ AMG/ QUI/ TET/ ANF/ IVF/ CARB/ POL	1
P4	7	PEN/ CEF/ QUI/ TET/ IVF/ CARB/ POL	2
P5	7	PEN/ AMG/ CEF/ IVF/ MON/ CARB/ POL	1
P6	7	PEN/ AMG/ CEF/ TET/ IVF/ MON/ POL	1
P7	6	PEN/ AMG/CEF/ TET/ MON/ POL	1
P8	6	PEN/ AMG/ CEF/ IVF/ MON/ POL	1
P9	6	PEN/ AMG/ QUI/ TET/ CARB/ POL	1
P10	6	PEN/ AMG/ QUI/ IVF/ ANF/ POL	1
P11	5	PEN/ AMG/ CEF/ MON/ POL	1
P12	5	PEN/ AMG/ CEF/ TET/ POL	2
P13	5	PEN/ AMG/ TET/ ANF/ POL	1
P14	5	PEN/ QUI/ GLI/ IVF/ POL	1
P15	5	PEN/ AMG/ TET/ CARB/ POL	1
P16	5	AMG/ QUI/ TET/ CARB/ POL	1
P17	5	PEN/ AMG/ CEF/ TET/ POL	1
P18	4	PEN/ CEF/ MON/ POL	2
P19	4	PEN/ AMG/ TET/ POL	1
P20	4	TET/ IVF/ AMG/ POL	1
P21	4	TET/ AMG/ CARB/ POL	1
P22	4	AMG/ QUI/ CARB/ POL	2
P23	4	PEN/ TET/ CARB/ POL	1
P24	4	AMG/ IVF/ TET/ POL	4
P25	4	PEN/ QUI/ TET/ POL	2
P26	4	AMG/ IVF/ CARB/ POL	2
P27	4	QUI/ TET/ CARB/ POL	6
P28	4	QUI/ TET/ ANF/ POL	1
P29	4	AMG/ TET/ CARB/ POL	1
P30	3	AMG/ TET/ POL	2
P31	3	PEN/ AMG/ POL	1
P32	3	PEN/ TET/ POL	6
P33	3	QUI/ CARB/ POL	6
P34	3	TET/ CARB/ POL	1
P35	3	TET/ IVF/ POL	3
P36	3	TET/QUI/ POL	4

*AMG* Aminoglycosides, *ANF* Amphenicols *CARB* Carbapenems *CEF* Cephalosporins, *GLI* Glycylcycline, *IVF* Folate pathway inhibitors, *MON* Monobactams, *PEN* Penicillins, *POL* Polymyxins, *QUI* Quinolones, *TET* Tetracyclines

### Molecular characterisation of resistance determinants

PCR screening for *mcr* variants revealed widespread presence of mobile polymyxin resistance genes. The *mcr-1* gene was detected in 99% (86/87) of resistant isolates, whilst *mcr*-5 was identified in a single *E. coli* isolate (1%). All *mcr*-positive isolates exhibited phenotypic resistance, with MIC values ranging from 8 to 64 µg/mL for *mcr*-1-positive isolates. Notably, the single *mcr*-1-positive *K. pneumoniae* isolate demonstrated the highest MIC value of 64 µg/mL, while the *mcr*-5-positive isolate showed an MIC of 32 µg/mL.

### Horizontal gene transfer capability

**Fig. 4:**
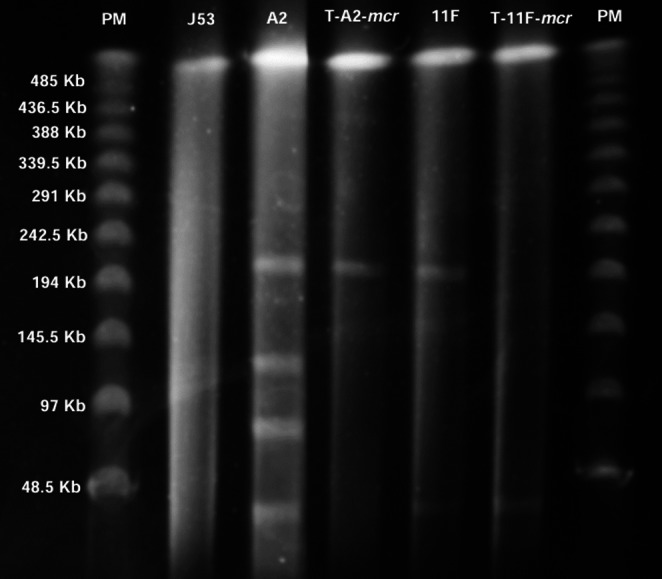
S1-nuclease pulsed-field gel electrophoresis (PFGE) analysis of *mcr-1*-carrying plasmids in transconjugants. Lanes show: PM, molecular weight marker; J53, recipient strain *E. coli* J53; 2 A, donor strain *E. coli* 2 A; T-2 A-mcr, transconjugant derived from *E. coli* 2 A carrying the *mcr-1* gene; 11 F, donor strain *K. pneumoniae* 11 F; T-11 F-mcr, transconjugant derived from *K. pneumoniae* 11 F carrying the *mcr-1* gene; PM, molecular weight marker. Single plasmids of approximately 194 kb and 48.5 kb were successfully transferred to the recipient strain in transconjugants T-2 A-mcr and T-11 F-mcr, respectively, confirming plasmid-mediated mobilisation of *mcr-1* resistance determinants

Conjugation experiments using *E. coli* 2 A and *K. pneumoniae* 11 F isolates as donors successfully demonstrated plasmid-mediated transfer of *mcr-1* gene to the recipient strain *E. coli* J53. Both transconjugants, designated T-2 A-mcr and T-11 F-mcr, exhibited increased polymyxin MICs from 0.25 µg/mL to 32 µg/mL. PCR screening confirmed that both carried the *mcr-1* gene, and MALDI-TOF MS identification verified them as *E. coli*. S1-nuclease PFGE analysis revealed transfer of single plasmids of approximately 194 kb in T-2 A-mcr and 48.5 kb in T-11 F-mcr, thus confirming the mobilisation capacity of these *mcr*-carrying elements (Fig. 4).

### Clonal diversity

**Fig. 5:**
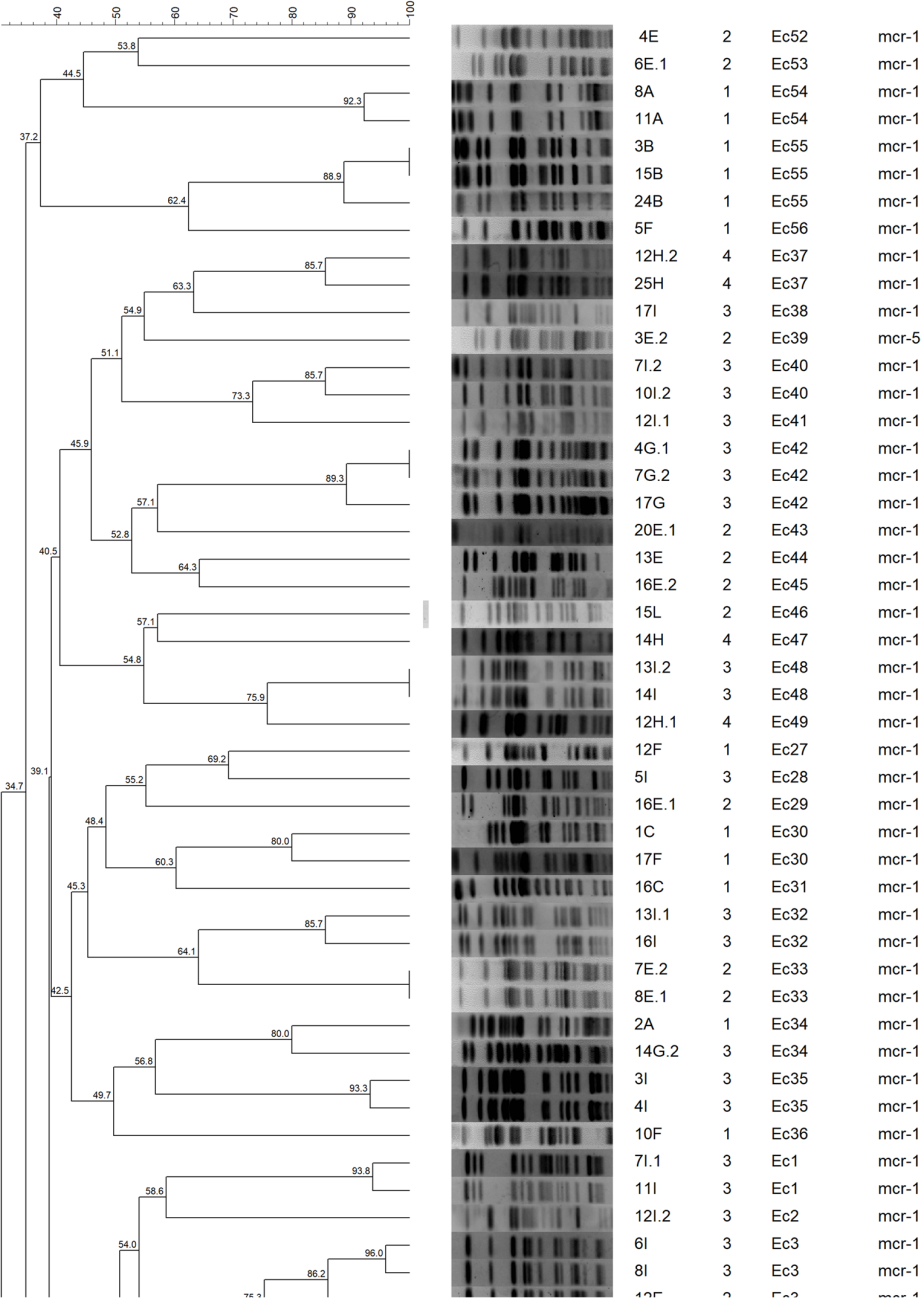

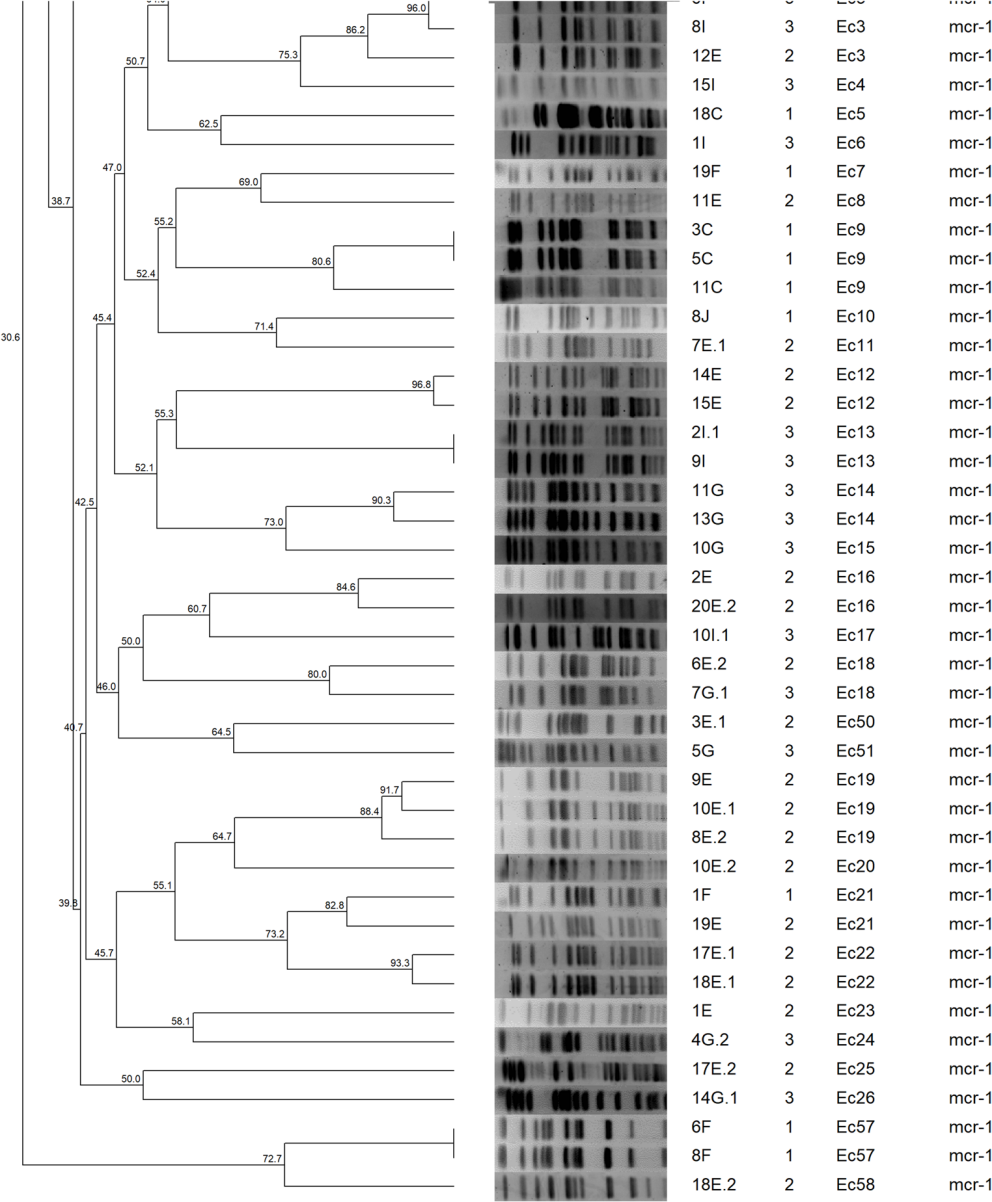
Pulsed-field gel electrophoresis (PFGE) dendrogram of 86 mcr-positive E. coli isolates from four broiler farms constructed using BioNumerics 6.6 software. Clonal groups (Ec1-Ec58) were defined at 80% similarity threshold. Numbers in the rightmost column indicate farm origin (1-4), and mcr gene variants are shown. The analysis revealed 58 distinct clonal groups, with 40.7% (35/86) of isolates displaying unique restriction patterns

PFGE analysis of the 86 *E. coli* isolates revealed remarkable genetic diversity, with 58 distinct clonal groups (Ec1-Ec58) identified at 80% similarity threshold. Notably, 40.7% (35/86) of isolates displayed unique restriction patterns, indicating substantial heterogeneity within the population. The remaining isolates formed small clusters, with 5 groups containing 3 isolates each and 18 groups comprising 2 isolates (Fig. 5).

**Fig. 6:**
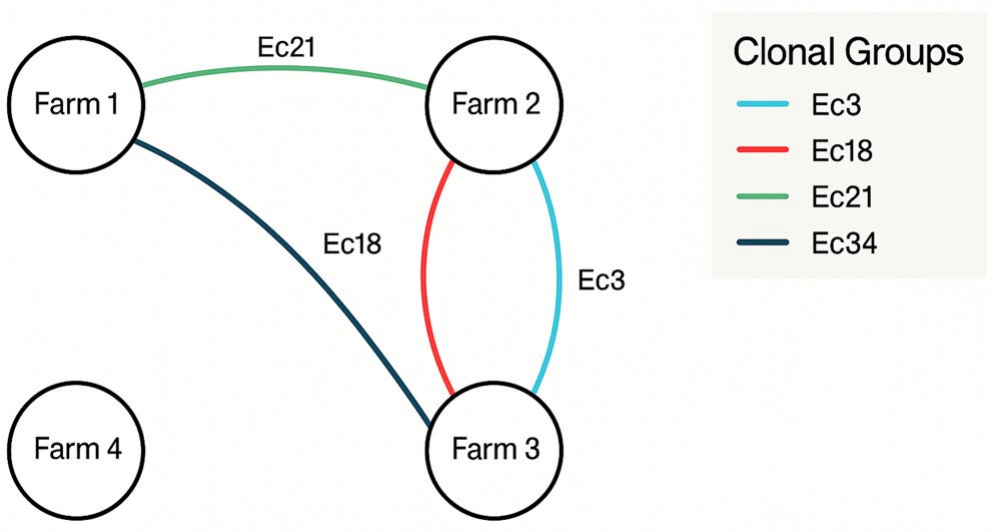
Inter-farm transmission patterns of *E. coli* clonal groups identified through PFGE analysis (created in Microsoft PowerPoint 365). Network diagram showing shared *E. coli* clonal groups (≥ 80% similarity) amongst 4 poultry farms. Connections indicate identical clones detected across multiple farms: Ec3 and Ec18 (Farms 2–3), Ec21 (Farms 1–2), and Ec34 (Farms 1–3). Farm 4 showed no shared clonal groups

Genetic diversity differed markedly between farms. Farm 2 presented the greatest heterogeneity, with 23 clonal groups identified among 29 isolates, followed by Farm 3 (22 groups from 32 isolates), Farm 1 (14 groups from 21 isolates) and Farm 4 (3 groups from 4 isolates). Notably, 4 clonal groups (Ec3, Ec18, Ec21 and Ec34) were detected on more than one farm, suggesting possible inter-farm transmission or a common source of contamination. Specifically, Ec3 and Ec18 occurred on Farms 2 and 3; Ec21 on Farms 1 and 2; and Ec34 on Farms 1 and 3 (Fig. 6).

The MLST results showed that isolate 3 C from farm 1 belonged to ST48 and isolate 14E from farm 2 to ST10; both fell into clonal complex 10 (CC10), suggesting a common lineage among these broiler-derived *E. coli* strains.

## Discussion

The persistence of polymyxin resistance in Brazilian poultry production systems, despite regulatory interventions, emerges as a central finding of this investigation. Our detection of 69.6% resistance frequency among enterobacterial isolates from Rio de Janeiro farms demonstrates that colistin resistance remains entrenched in poultry-associated bacterial populations three to four years after the 2016 regulatory ban on its use as a growth promoter [3]. This observation aligns with global patterns where antimicrobial resistance, once established, tends to persist even after selective pressure removal, particularly when resistance determinants impose minimal fitness costs on bacterial hosts [21].

The predominance of *E. coli* (99%) among resistant isolates corroborates international findings positioning this species as the primary reservoir for mobile polymyxin resistance determinants in livestock environments [22]. When examining the broader Brazilian context, our findings contribute to a complex picture of resistance patterns that appear to vary considerably across different time periods, geographical regions, and study methodologies. Previous multi-state surveillance conducted prior to the polymyxin ban reported 40% resistance rates [11], while a regional study from Rio de Janeiro during 2015–2016 documented substantially higher frequencies of 95.3% [12]. The resistance levels observed in our investigation may reflect the complex interplay of regional factors, including local antimicrobial usage patterns, farm-specific management practices, and varying biosecurity implementation strategies across different poultry production systems.

The molecular epidemiology of resistance mechanisms provides compelling evidence for the dominance of plasmid-mediated resistance. The overwhelming prevalence of *mcr-1* (99% of resistant isolates) mirrors global surveillance data identifying this variant as the most widely disseminated mobile polymyxin resistance determinant [10]. Interestingly, our detection of a single *mcr*-5-positive isolate represents one of the few reports of this variant in Brazilian poultry, contrasting with its higher prevalence in European livestock systems [23]. The absence of other *mcr* variants in this study should be interpreted with caution, as only *mcr-1*, *mcr-5*, and *mcr-9* were investigated by multiplex PCR. Therefore, the circulation of additional *mcr* alleles in the regional poultry microbiome cannot be excluded.

Conjugation experiments provided evidence of horizontal transferability for *mcr*-carrying plasmids, with successful mobilisation of genetic elements ranging from 48.5 to 194 kb. These findings substantiate concerns regarding the potential for resistance gene dissemination across bacterial populations and species barriers, as previously demonstrated in clinical and environmental settings [24]. The successful transfer from both *E. coli* and *K. pneumoniae* donors to the recipient strain underscores the promiscuous nature of *mcr*-carrying plasmids and their capacity to traverse taxonomic boundaries. Notably, our group has previously provided pioneering evidence in Brazil of inter- and intraspecies mobilization of the *mcr* gene between clinical *E. coli* and *K. pneumoniae* isolates [25, 26], underscoring the One Health dimension of the problem. When considered alongside the present findings in poultry isolates, these data highlight the interconnectedness of human, animal, and environmental reservoirs in the dissemination of plasmid-mediated colistin resistance.

The multidrug resistance landscape reveals a concerning panorama, with 75% of our isolates classified as MDR according to internationally accepted criteria [5]. This proportion exceeds resistance rates reported in comparable studies from other middle-income countries, though it remains consistent with intensive poultry production systems where antimicrobial selection pressure remains substantial [27]. The co-occurrence of resistance to critically important antimicrobials, particularly nalidixic acid (60.9%) and tetracycline (58.6%), despite the prohibition of quinolones and tetracyclines as performance-enhancing additives since 2009 in Brazil, may suggest either continued off-label usage or the persistence of resistance determinants in bacterial populations through co-selection mechanisms [28]. The observation of higher ertapenem resistance relative to imipenem and meropenem may reflect permeability-related mechanisms, notably porin loss (OmpK35/OmpK36) and co-occurring ESBL/AmpC enzymes, that selectively elevate ertapenem MICs while leaving imipenem/meropenem susceptible [29]. This pattern has been described previously and highlights the importance of investigating porin and β-lactamase contributions when interpreting non-uniform carbapenem susceptibility profiles.

Genetic diversity analysis through PFGE revealed extensive heterogeneity among resistant *E. coli* populations, with 59 distinct clonal groups identified among 86 isolates. This polyclonal structure argues against the hypothesis of clonal expansion and instead supports the theory that resistance persistence results from sustained selective pressure acting upon genetically diverse bacterial communities [30]. The identification of shared clonal groups across different farms suggests potential inter-farm transmission routes, possibly mediated through contaminated feed, water sources, equipment, or personnel movement, emphasising the interconnected nature of modern agricultural systems. The absence of a predominant clone suggests that polymyxin resistance in this population results from selective pressure rather than clonal expansion of a specific epidemic strain, highlighting the widespread nature of *mcr* gene dissemination across diverse *E. coli* lineages in Brazilian poultry production systems.

The geographic clustering of resistance determinants within a relatively confined region raises important questions about the environmental persistence and ecological fitness of *mcr*-carrying bacteria. Recent investigations have demonstrated the capacity of these organisms to survive in diverse environmental matrices, including soil, water, and organic waste, potentially serving as repositories for continued reintroduction into production systems [31]. This environmental dimension underscores the complexity of resistance control strategies and the need for comprehensive, ecosystem-based approaches to antimicrobial stewardship.

From a One Health perspective, our findings highlight concerning implications for food safety and public health. The detection of transferable resistance determinants in food-producing animals creates direct pathways for resistance gene flow into human-associated bacterial populations through occupational exposure, foodborne transmission, and environmental contamination [13].

Preliminary molecular typing by MLST analysis of *mcr*1-positive *E. coli* isolates from our study showed the presence of STs of considerable epidemiological significance, including ST10 and ST48, high risk clones detected in human, food production animals, fresh produce, and environmental isolates. The detection of these pandemic clones in our poultry isolates is particularly concerning given that CC10 has demonstrated remarkable adaptability through lateral gene transfer mechanisms and has been implicated in the global dissemination of clinically relevant resistance genes, including those encoding resistance to polymyxin, cephalosporins, carbapenems, and fluoroquinolones [32–34].

Finally, our study provides insights into the long-term effectiveness of policy-based approaches to antimicrobial resistance control. Whilst the ban on polymyxin as a growth promoter represents a significant regulatory achievement, the persistence of resistance suggests that additional interventions may be required to achieve meaningful reductions in resistance prevalence. These might include enhanced surveillance systems, improved biosecurity protocols, alternative growth promotion strategies, and more stringent enforcement mechanisms.

## References

[1] Velazquez-meza ME et al (2022) Antimicrobial resistance: one health approach. Vet World 15(3):743–749 35497962 10.14202/vetworld.2022.743-749 PMC9047147

[2] Ponzo E et al (2024) The Antimicrobial Resistance Pandemic Is Here: Implementation Challenges and the Need for the One Health Approach. Hygiene 4(3):297–316

[3] Rabello RF et al (2020) Antimicrobial Resistance in Farm Animals in Brazil: An Update Overview. Animals: open access J MDPI 10(4):552–526 10.3390/ani10040552 PMC7222418 32224900

[4] kathayat D et al (2021) Avian pathogenic escherichia coli (APEC): an overview of virulence and pathogenesis factors, zoonotic potential, and control strategies. Pathogens (Basel Switzerland) 10(4):467–412 33921518 10.3390/pathogens10040467 PMC8069529

[5] Magiorakos A-P et al (2012) Multidrug-resistant, extensively drug-resistant and pandrug-resistant bacteria: an international expert proposal for interim standard definitions for acquired resistance. Clin Microbiol Infection: Official Publication Eur Soc Clin Microbiol Infect Dis 18(3):268–281 10.1111/j.1469-0691.2011.03570.x 21793988

[6] Platell JL et al (2011) Multidrug-resistant extraintestinal pathogenic Escherichia coli of sequence type ST131 in animals and foods. Vet Microbiol 153(1–2):99–108 21658865 10.1016/j.vetmic.2011.05.007

[7] Poirel L, Jayol A, Nordmann P (2017) Polymyxins: antibacterial activity, susceptibility testing, and resistance mechanisms encoded by plasmids or chromosomes. Clin Microbiol Rev 30(2):557–596 28275006 10.1128/CMR.00064-16 PMC5355641

[8] Kempf I, Jouy E, Chauvin C (2016) Colistin use and colistin resistance in bacteria from animals. Int J Antimicrob Agents 48(6):598–606 27836380 10.1016/j.ijantimicag.2016.09.016

[9] liu Y-Y et al (2016) Emergence of plasmid-mediated colistin resistance mechanism MCR-1 in animals and human beings in China: a microbiological and molecular biological study. Lancet Infect Dis 16(2):161–168 26603172 10.1016/S1473-3099(15)00424-7

[10] Valiakos G, Kapna I (2021) Colistin resistant mcr genes prevalence in livestock animals (swine, bovine, poultry) from a multinational Perspective. Syst Rev Vet Sci 8(11):265 10.3390/vetsci8110265 PMC8619609 34822638

[11] Fernandes MR et al (2016) Silent dissemination of colistin-resistant *Escherichia coli* in South America could contribute to the global spread of the mcr-1 gene. Euro Surveillance: Bull Europeen Sur Les Maladies Transmissibles = Eur Commun Disease Bull 21(17):28 10.2807/1560-7917.ES.2016.21.17.30214 27168587

[12] Barbieri NL et al (2021) mcr-1 identified in fecal *Escherichia coli* and avian pathogenic *E. coli* (APEC) from Brazil. Front Microbiol 12:659613 33959114 10.3389/fmicb.2021.659613 PMC8093808

[13] Lammie SL, Hughes JM (2016) Antimicrobial resistance, food safety, and one health: the need for convergence. Annual Rev Food Sci Technol 7:287–312 26772408 10.1146/annurev-food-041715-033251

[14] Conceição-Neto OC (2022) Polymyxin resistance in clinical Isolates of K. pneumoniae in Brazil: update on molecular mechanisms, clonal dissemination and relationship with KPC-producing strains. Front Cell Infect Microbiol 12:15 10.3389/fcimb.2022.898125 PMC9334684 35909953

[15] Rebelo AR et al (2018) Multiplex PCR for detection of plasmid-mediated colistin resistance determinants, mcr-1, mcr-2, mcr-3, mcr-4 and mcr-5 for surveillance purposes. Euro Surveillance: Bulletin Europeen Sur Les Maladies Transmissibles = European. Commun Disease Bull 23(6):17–00672 10.2807/1560-7917.ES.2018.23.6.17-00672 PMC5824125 29439754

[16] Li R et al (2017) Complete genetic analysis of plasmids carrying mcr-1 and other resistance genes in an *Escherichia coli* isolate of animal origin. J Antimicrob Chemother 72(3):696–699 27999050 10.1093/jac/dkw509

[17] Pontes LDAS et al (2021) ) Letter to the editor: *Escherichia fergusonii* Harboring IncHI2 plasmid containing mcr-1 gene-a novel reservoir for colistin resistance in Brazil. Microbial Drug Resistance (Larchmont, N.Y.) 27(5):721–725 10.1089/mdr.2020.0041 33001761

[18] Ribot EM et al (2006) Standardization of pulsed-field gel electrophoresis protocols for the subtyping of Escherichia coli O157:H7, Salmonella, and Shigella for PulseNet. Foodborne Pathog Dis 3(1):59–67 16602980 10.1089/fpd.2006.3.59

[19] Kalanxhi E et al (2021) Confidence interval methods for antimicrobial resistance surveillance data. Antimicrob Resist Infect Control 10,(1):91–99 34108041 10.1186/s13756-021-00960-5 PMC8191092

[20] Magiorakos A-P et al (2012) mar. Multidrug-resistant, extensively drug-resistant and pandrug-resistant bacteria: an international expert proposal for interim standard definitions for acquired resistance. Clinical Microbiology and Infection: The Official Publication of the European Society of Clinical Microbiology and Infectious Diseases. 18(3):268–281 10.1111/j.1469-0691.2011.03570.x 21793988

[21] Andersson DI, Hughes D (2014) Microbiological effects of sublethal levels of antibiotics. Nat Rev Microbiol 12:465–478 24861036 10.1038/nrmicro3270

[22] Luo Q, Wang Y, Xiao Y (2020) Prevalence and transmission of mobilized colistin resistance (mcr) gene in bacteria common to animals and humans. Biosaf Health 2(2,):71–78

[23] Borowiak M et al (2017) Identification of a novel transposon-associated phosphoethanolamine transferase gene, mcr-5, conferring colistin resistance in d-tartrate fermenting Salmonella enterica subsp. enterica serovar Paratyphi B. J Antimicrob Chemother 72(12):3317–3324 28962028 10.1093/jac/dkx327

[24] Carroll LM et al (2019) Identification of novel mobilized colistin resistance gene mcr-9 in a multidrug-resistant, colistin-susceptible salmonella enterica serotype typhimurium isolate. mBio 10(3):07 10.1128/mBio.00853-19 PMC6509194 31064835

[25] Aires CAM et al (2017) Emergence of the plasmid-mediated mcr-1 gene in clinical KPC-2-producing *Klebsiella pneumoniae s*equence type 392 in Brazil. Antimicrob. Agents Chemo. 61(7):10.1128/aac.00317-17 10.1128/AAC.00317-17 PMC5487683 28438940

[26] Conceição-Neto, OC et al (2017) Detection of the plasmid-mediated mcr-1 gene in clinical KPC-2-producing Escherichia coli isolates in Brazil. Int J Antimicrob Agents 50(2):282–284 28579456 10.1016/j.ijantimicag.2017.05.003

[27] Brower CH et al (2017) The Prevalence of extended-spectrum beta-lactamase-producing multidrug-resistant Escherichia Coli in poultry Chickens and variation according to farming practices in Punjab, India. Environ Health Perspect 125(7):077015 28749780 10.1289/EHP292 PMC5744676

[28] Korb A et al (2015) mar. Tipagem molecular e resistência aos antimicrobianos em isolados de Escherichia coli de frangos de corte e de tratadores na Região Metropolitana de Curitiba, Paraná. Pesq Vet Bras 35(3):258–264

[29] Tsai Y-K et al (2013) nov. Single or in combination antimicrobial resistance rechanisms of klebsiella pneumoniae contribute to varied susceptibility to different carbapenems. PLoS ONE 8(11):e79640 24265784 10.1371/journal.pone.0079640 PMC3827147

[30] Liu B-T et al (2017) High incidence of Escherichia coli strains coharboring mcr-1 and blaNDM from Chickens. Anti Agents Chemo 61(3):e02347–e02316 10.1128/AAC.02347-16 PMC5328528 28069644

[31] Wang R et al (2018) The global distribution and spread of the mobilized colistin resistance gene mcr-1. Nat Commun 9(1):1179 10.1038/s41467-018-03205-z PMC5862964 29563494

[32] Pérez-Etayo L, González D, Vitas AI, Clonal (2022) Complexes 23, 10, 131 and 38 as genetic markers of the environmental spread of extended-spectrum β-lactamase (ESBL)-Producing *E. coli*. Antibiotics 11(11):1465 10.3390/antibiotics11111465 PMC9686695 36358120

[33] Sanz MB et al (2022) Carbapenemase-producing extraintestinal pathogenic *Escherichia coli* From Argentina: clonal diversity and predominance of hyperepidemic clones CC10 and CC131. Front Microbiol 13:18 10.3389/fmicb.2022.830209 PMC8971848 35369469

[34] De Koster S et al (2023) Genetic characterization of ESBL-producing and ciprofloxacin-resistant Escherichia coli from Belgian broilers and pigs. Front. Microbiol. 14(6) 10.3389/fmicb.2023.1150470 PMC10116946 37089550

